# CT severity score: an imaging biomarker to estimate the severity of COVID-19 pneumonia in vaccinated and non-vaccinated population

**DOI:** 10.1186/s43055-022-00768-2

**Published:** 2022-04-12

**Authors:** Balasubramanian Gurumurthy, Sudha Kiran Das, Sachin Shetty, Rakesh Chowkalli Veerabhadrappa, Sai Siddartha Kosinepalli, Swathi Hassan Dharamaraju

**Affiliations:** 1 Department of Radiology, JSS Hospital, JSS Academy of Higher Education and Research, Ramachandra Agrahara, Mysore, Karnataka 570004 India; 2 Department of Speech and Hearing, Manipal College of Health Professions, Manipal Academy of Higher Education, Manipal, Karnataka India

**Keywords:** Covaxin, COVID-19, Covishield, CT severity score

## Abstract

**Background:**

In India, two vaccines received emergent use authorization, namely Covishield (a brand of the Oxford—Astra Zeneca vaccine manufactured by the Serum institute of India) and Covaxin (developed by Bharat Biotech) against COVID-19 disease. Chest CT is an objective way to assess the extent of pulmonary parenchymal involvement. This study aims to estimate the disease severity and outcome due to COVID-19 among vaccinated and non-vaccinated symptomatic patients and compare the same in Covishield versus Covaxin recipients using CT severity score.

**Results:**

A total of 306 patients were retrospectively evaluated. The mean age was 62.56 ± 8.9 years, and males [*n*-208 (67.97%)] were commonly affected. Of 306 patients, 143 were non-vaccinated (47%), 124 were partially vaccinated (40%), and 39 were completely vaccinated (13%). CT severity scores were reduced in both Covishield and Covaxin recipients in comparison with the non-vaccinated group [*χ*^2^ (2) = 16.32, *p* < 0.001]. There is a reduction in LOS among the vaccinated group, predominantly among the Covishield recipients.

**Conclusion:**

Vaccination confers protection from severe SARS-CoV2 infection and is associated with an overall reduction in mortality.

## Background

Ever since the Coronavirus disease 2019 (COVID-19) was caused by a severe acute respiratory syndrome, coronavirus 2 (SARS-CoV-2) was declared a pandemic by the World Health Organization (WHO) on March 11, 2020, the disease has continued to spread unabated globally, resulting in widespread morbidity and mortality [1]. Ever-changing information about the SARS-CoV2 infection and multi-organ involvement was confounded by the changing landscape of therapeutic options. Safe and effective vaccines have been considered a game-changing tool and critical in ending the COVID-19 pandemic. Over 184 vaccines are under pre-clinical development, 108 vaccines under clinical development, 19 are in Phase III clinical trials, and eight of them have ended Phase III [2]. In India, two vaccines initially received approval for emergent use by the Ministry of Health (MoH) on January 16, 2021 in a phased manner; namely, (1) Oxford—Astra Zeneca vaccine [ChAdOx1 nCoV-19 vaccine (AZD1222)], a chimpanzee adenoviral vectored vaccine with full-length SARS-CoV-2 spike insert developed at the University of Oxford (Oxford, UK) and manufactured by the Serum institute of India with brand name Covishield and (2) Covaxin (BBV152), a whole-virion inactivated SARS-CoV-2 vaccine formulated with a toll-like receptor (TLR) 7/8 agonist molecule adsorbed to alum (Algel-IMDG) developed by Bharat Biotech, India [3, 4]. Covishield has shown the efficacy of 76.0% at preventing symptomatic COVID-19 following the first dose and 81.3% after the second dose. In contrast, Covaxin following two doses showed vaccine efficacy of 77.8% against symptomatic COVID-19 disease and the higher efficacy of 93.4% against severe COVID-19 disease [3, 5].

HRCT chest imaging plays a pivotal role in early disease detection, monitoring the course of COVID-19 pneumonia, and also works as an indicator of disease severity and possible outcome [6]. The Chest CT severity score (CT-SS) is a proven tool for the initial evaluation of COVID-19 patients as it positively correlates with the inflammatory markers indicative of disease severity. Studies have proven its efficacy to predict severe disease and the need for ICU admission [7]. This study aims to estimate the disease severity and outcome secondary to SARS-CoV2 infection among vaccinated and non-vaccinated symptomatic patients. It also aims to compare the efficacy of Covishield versus Covaxin using CT-SS in correlation to the length of hospital stay and outcome.

## Methods

### Study design and data collection

This retrospective observational study was conducted for 4 months between March 1 and June 30, 2021. The study included *confirmed* COVID-19 pneumonia cases and *probable* COVID-19 cases (COVID-19 like syndrome) who underwent HRCT thorax. The *confirmed* and *probable cases* of SARS-CoV-2 infection are defined as per WHO COVID-19 case definition [8]. The disease severity among vaccinated, partially vaccinated, and non-vaccinated patients was evaluated using CT severity scores. The HRCT images were assessed using the institutional Picture Archiving and Communication Systems (PACS) database system. We also collected the clinical data of the cohort from the institutional electronic medical record system. The Institutional Ethics committee approval was obtained for this study.

### Selection criteria

The study conducted herein was done during the second phase of the vaccination drive in India, which started on March 1st, 2021, where only people aged above 60 years and people aged between 45 and 60 years with comorbidities were eligible. After that, from April 1st, people aged 45 years were also considered for vaccination. Hence, the study included confirmed and probable cases of COVID-19 disease who underwent HRCT chest imaging and whose vaccination status was known. Patients below 50 years, patients whose vaccination status was not known, and patients who got discharged against medical advice were excluded.

### HRCT imaging protocol

The HRCT for the patients was performed using a 128-slice MDCT scanner (Ingenuity core 128 v3.5.7.25001; Philips healthcare). Patients were placed in a supine position with a single breath-hold. Scanning parameters were scan direction (cranio-caudally), tube voltage (120 kV), tube current (250 mA), slice collimation (64 × 0.625 mm), width (0.625 × 0.625 mm), pitch (1), rotation time (0.5 s) and scan time (12.06 s). Images were reconstructed with a slice thickness of 0.5 or 1.5 mm and an interval of 0.5 or 1.5 mm, respectively.

### HRCT imaging interpretation

The images were evaluated by a professor, assistant professor, and a senior resident in radiology with experience of 18 years, 7 years, and 5 years, respectively. The CT features of the cohort were independently assessed by the evaluators using both axial CT images and multi-planar reconstruction images. The severity of the disease was assessed using CT severity score (total score out of 25) and categorized into mild (score-< 7), moderate (score 7–18), and severe (score > 18) [6, 9, 10]. The professor and assistant professor were blinded to the vaccination status and outcome of the cohort.

### Statistical analysis

The raw scores were tabulated and subjected to statistical analysis using IBM SPSS 20. Descriptive statistics of patients’ demographics and clinical results were reported as a number and relative frequencies. The CT severity scores among the cohort did not follow a normal distribution (Shapiro–Wilk test); hence, nonparametric statistics were carried out. Kruskal Wallis and Mann–Whitney *U* test were performed to check if the descriptive difference observed was statistically significant.

## Results

### Epidemiology and clinical features

A total of 407 confirmed and probable cases of COVID-19 underwent HRCT chest between March 1 and June 30, 2021. One hundred one were excluded from the cohort as per exclusion criteria stated before. Eventually, 306 patients were included in the study and evaluated retrospectively. Out of 306 cases, 267 were *confirmed* cases, and 39 were *probable* cases. The demographic information pertaining to age, gender, presenting symptoms/complaints, presence of comorbidities/risk factors, vaccination status, dose and type of vaccination, CT severity score, duration of hospital stay among admitted patients, and the disease outcome (alive or died) are as below.

The mean age was 62.56 ± 8.9 years ranging from 50 to 99 years. The age of the cohort was further classified into five groups: 50–59, 60–69, 70–79, 80–89, and 90–99, as depicted in Table 1. The age group (50–59) had the highest cases with a total of 126 cases (41.1%). Males [*n*-208 (67.97%)] were affected more compared to females [*n*-98 (32.02%).]

**Table 1:** Age distribution of the cohort

	Total number (out of 306)	Percentage (%)
50–59	126	41.1
60–69	114	37.3
70–79	50	16.3
80–89	15	5.0
90–99	1	0.3

The most common presenting symptoms were fever [*n*-189, (61.7%)] and cough [*n*-178, (58.1%)]. Other symptoms were hemoptysis, generalized weakness, dyspnea, myalgia, diarrhea, and others, and the frequencies of the same are depicted in Table 2.

**Table 2:** Presenting symptoms of the cohort

Symptom	Total number	Percentage (out of 306)
Fever	189	61.7
Cough	178	58.1
Dyspnea	47	15.3
Generalized weakness	43	14.0
Myalgia	37	12.0
Hemoptysis	2	0.6
URTI symptoms	13	4.2
Diarrhea	20	6.5
Vomiting	7	2.2
Easy fatigability	23	7.5
Pain abdomen	4	1.3
Chest pain	1	0.3
Headache	11	3.5
Loss of smell/anosmia	5	1.6
Loss of taste	2	0.6
Focal neurological deficit	4	1.3
Loss of appetite	4	1.3
Seizure	1	0.3
Hematuria	1	0.3
Otalgia	1	0.3

The comorbidities/risk factors considered were hypertension, diabetes mellitus, cardiovascular disease, cerebrovascular diseases, chronic obstructive pulmonary disease, and others are depicted in Table 3. Comorbidities/risk factors were found in 259/306 patients (84.6%). The most common associations were diabetes mellitus [*n*-170, (55.5%)] and hypertension [*n*-154, (50.3%)].

**Table 3:** Comorbidities/risk factors of the cohort

Comorbidities	Total number	Percentage (out of 306)
Cardiovascular disease	30	9.8
Cerebrovascular disease	3	0.9
Chronic Kidney disease	10	3.2
Chronic obstructive pulmonary disease	10	3.2
Diabetes	170	55.5
Hypertension	154	50.3
Hypothyroidism	19	6.2
Immunocompromised	1	0.3
Others	7	2.2

**Fig. 1:**
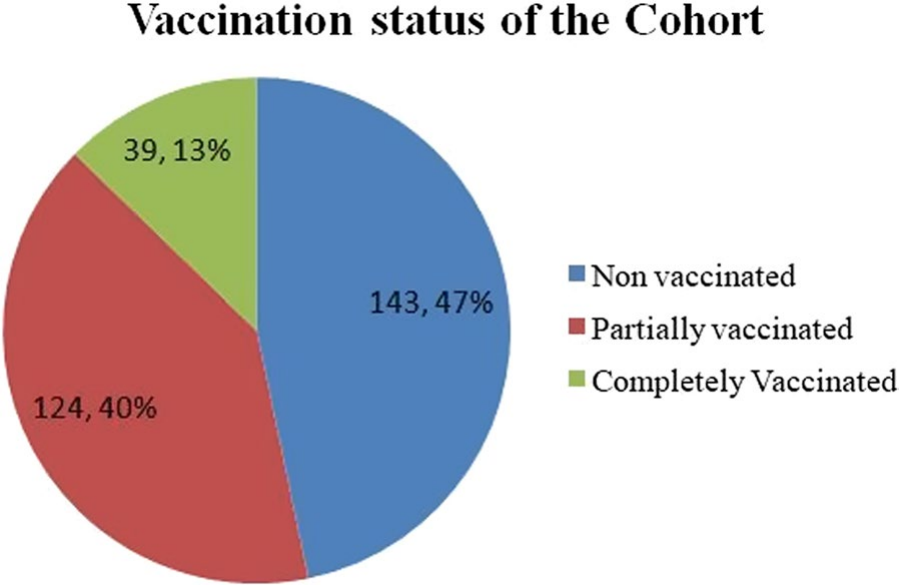
Vaccination status of the cohort

The vaccination status of the cohort is depicted in Fig. 1. Among 124 partially vaccinated patients, 87 were Covishield recipients (70.1%), and 37 were Covaxin recipients (29.9%). Among 39 completely vaccinated patients, 20 were Covishield recipients (51.2%), and 19 were Covaxin recipients (48.2%).

### Vaccination and CT severity score

**Fig. 2:**
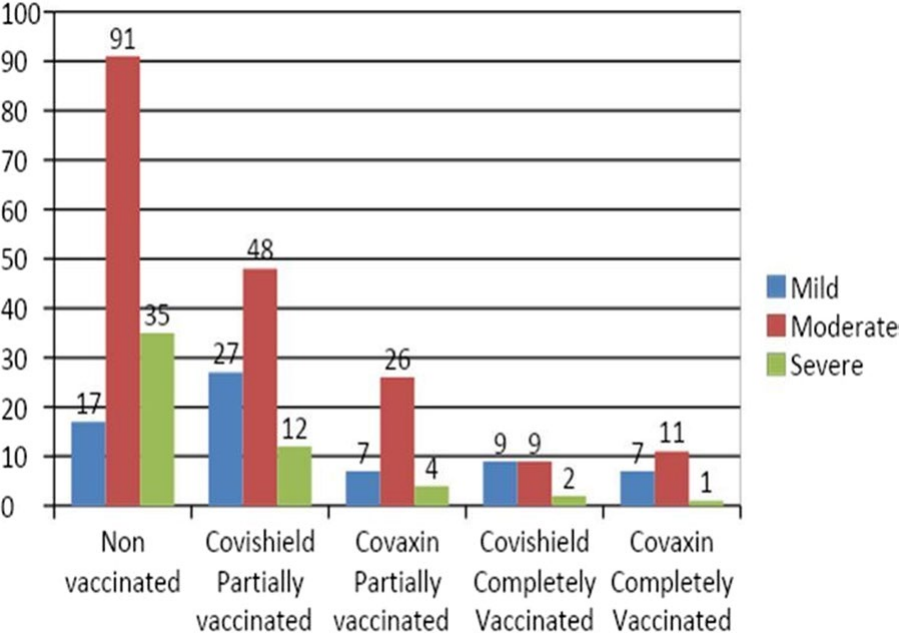
CT severity of the cohort

**Fig. 3:**
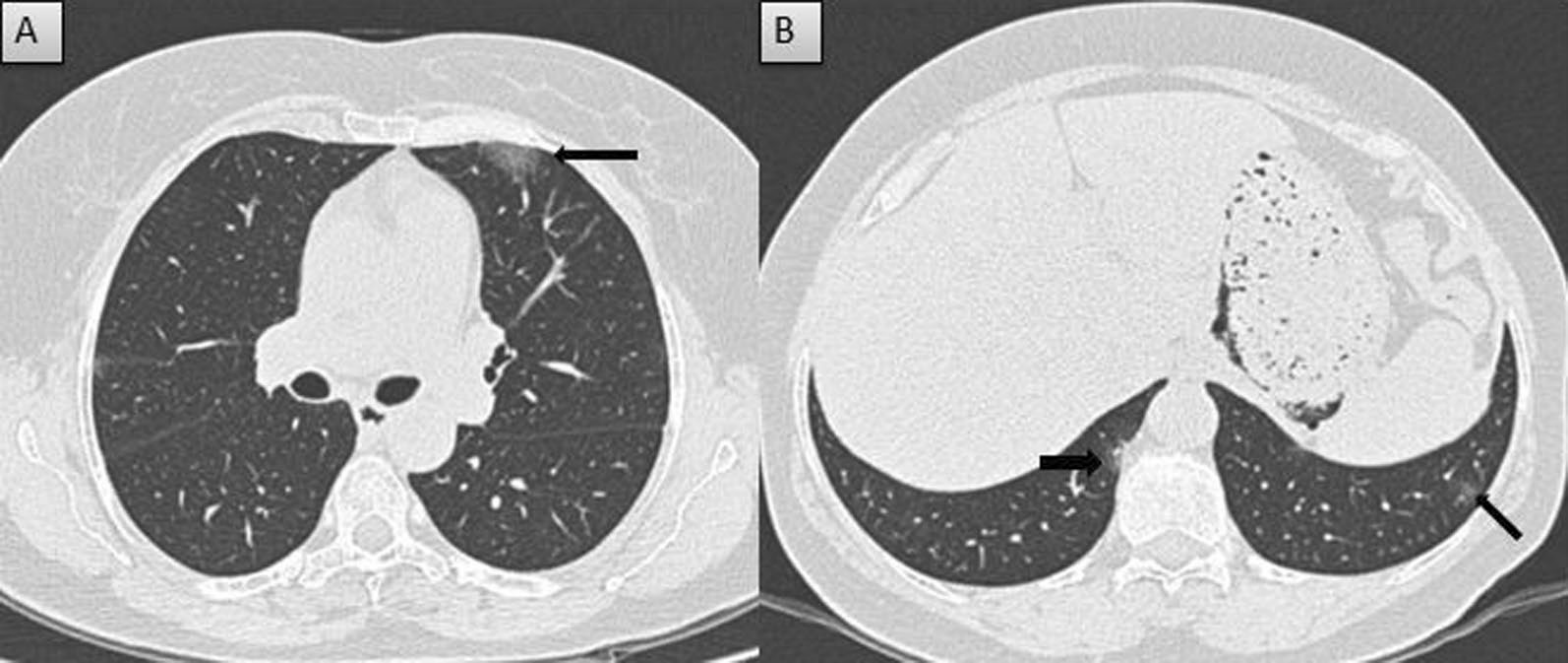
Mild severity in completely vaccinated patient: **A** and **B** Axial CT thorax (lung window) of a 59-year-old COVID pneumonia patient who had received two doses of Covid vaccine shows patchy peripheral ground glass opacities with CT severity score of 3/25 (mild severity)

**Fig. 4:**
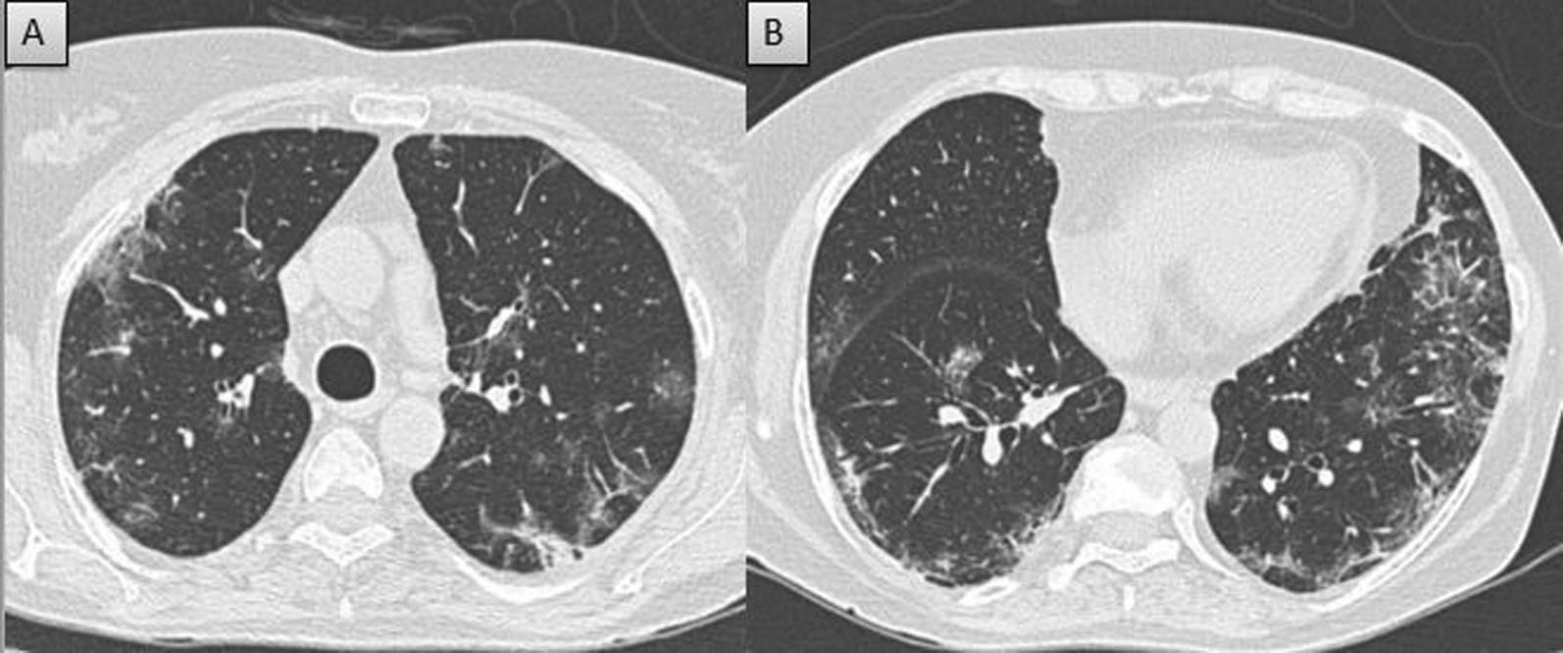
Moderate severity in partially vaccinated patient: **A** and **B** Axial CT thorax (lung window) of a 61-year-old COVID pneumonia patient who had received single dose of Covid vaccine shows diffuse peripheral patchy ground glass opacities and interstitial thickening with CT severity score of 14/25 (moderate severity)

**Fig. 5:**
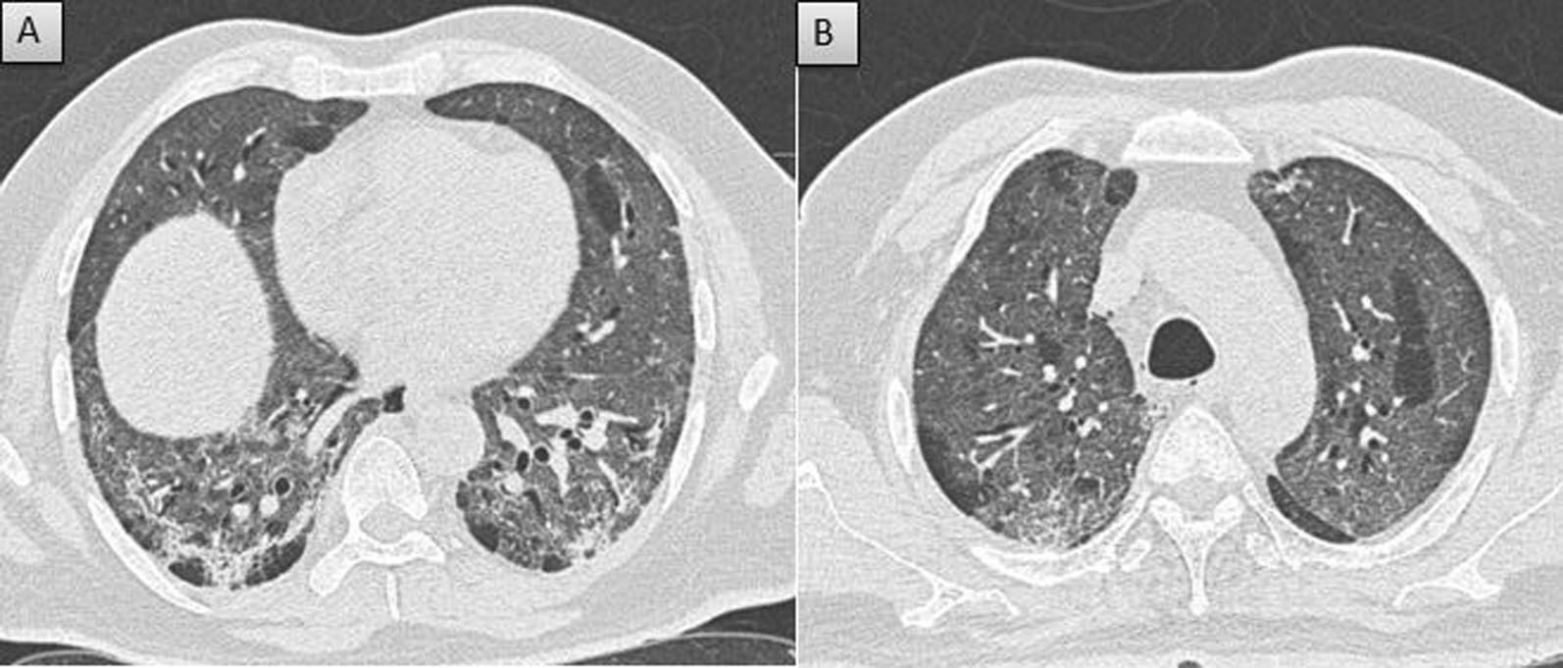
Severe severity in non-vaccinated patient: **A** and **B** Axial CT thorax (lung window) of a 57-year-old non-vaccinated COVID pneumonia patient shows diffuse confluent ground glass opacities with CT severity score of 22/25 (severe severity)

The CT severity score of the cohort is depicted in Table 4 and Fig. 2. It was observed that the disease severity in terms of CT severity score was reduced among the patients with vaccination irrespective of the dose compared with the non-vaccinated group. Also, the CT severity was reduced in both Covishield recipients (median-11) and Covaxin recipients (median-11) when compared to the non-vaccinated group (median-13). To check for the difference in the CT scores between Covishield, Covaxin, and non-vaccinated groups, the Kruskal Wallis test was performed. The results revealed a statistically significant difference among the three groups [*χ*^2^ (2) = 16.32, *p* < 0.001]. Further, the Mann–Whitney *U* test was performed, and the results indicated a statistically significant difference in CT scores between the non-vaccinated and Covishield group (/*Z*/ = 3.84, *p* < 0.001) and non-vaccinated and Covaxin group (/*Z*/ = 2.49, *p* < 0.001). Results indicate that the CT scores observed in vaccinated groups were significantly reduced compared to the non-vaccinated group. However, there was no statistically significant difference between Covishield and Covaxin recipients (/*Z*/ = 0.66, *p* = 0.50). Similarly, there was no significant difference between partially vaccinated (single dose) and completely vaccinated (double dose) group among Covishield (/*Z*/ = 1.18, *p* = 0.238), and Covaxin recipient (/*Z*/ = 1.08, *p* = 0.278). A few examples of the CT severity score of the cohort are depicted in Figs. 3, 4 and 5.

**Table 4:** CT severity of the cohort

Vaccination status	CT severity score		
	Mild (score-< 7)	Moderate (score 7–18)	Severe (> 18)
Non vaccinated (out of 143)	17 (11.9%)	91 (63.6%)	35 (24.5%)
Covishield partially vaccinated (out of 87)	27 (31.0%)	48 (55.2%)	12 (13.8%)
Covishield completely vaccinated (out of 20)	9 (45%)	9 (45%)	2 (10%)
Covaxin partially vaccinated (out of 37)	7 (18.9%)	26 (70.3%)	4 (10.8%)
Covaxin completely vaccinated (out of 19)	7 (36.9%)	11 (57.9%)	1 (5.2%)

### Vaccination and length of hospital stay (LOS)

Regarding the length of hospital stay (LOS), the descriptive statistics were obtained for 234 patients whose clinical outcomes were good and discharged from the hospital. Patients who succumbed were not included in the evaluation for LOS. The LOS for the non-vaccinated group had a median was 11, with 25th (Q1) and 75th (Q2) percentile being 7 and 15, respectively, and with a mean of 12.08 with an SD of 6.39. For the Covaxin group, the median was 9 with Q1 and Q2 of 6.75 and 13, respectively, and the mean score was 10.21 with an SD of 4.76. The Covishield group LOS had a median of 9, with Q1 and Q2 being 7 and 11, respectively, with a mean score of 9.36 (SD = 3.42). To check for the difference in the LOS between non-vaccinated, Covaxin, and Covishield groups, the Kruskal Wallis test was carried out. The results indicated a statistically significant difference among the three groups [*χ*^2^ (2) = 8.52, *p* = 0.01]. Further, the Mann–Whitney *U* test was performed, and the results revealed a significant difference in LOS between Covishield and the non-vaccinated group (/*Z*/ = 2.86, *p* = 0.004). Furthermore, the Mann–Whitney *U* test results showed no significant difference between the first and second dose group (/*Z*/ = 0.11, *p* = 0.911). Similarly, there was no statistically significant difference between non-vaccinated and Covaxin group (/*Z*/ = 1.65, *p* = 0.09) and Covishield and Covaxin recipients (/*Z*/ = 0.44, *p* = 0.65). Overall there is a reduction in LOS among the vaccinated group, predominantly among the Covishield recipients.

Spearmen rank correlation coefficient analysis was conducted to check any correlation between the CT score and LOS. The results revealed a significant positive moderate correlation (*ρ* = 0.325, *p* < 0.001). The findings indicate that as the CT score increases, so does the LOS.

### Vaccination and outcome

**Fig. 6:**
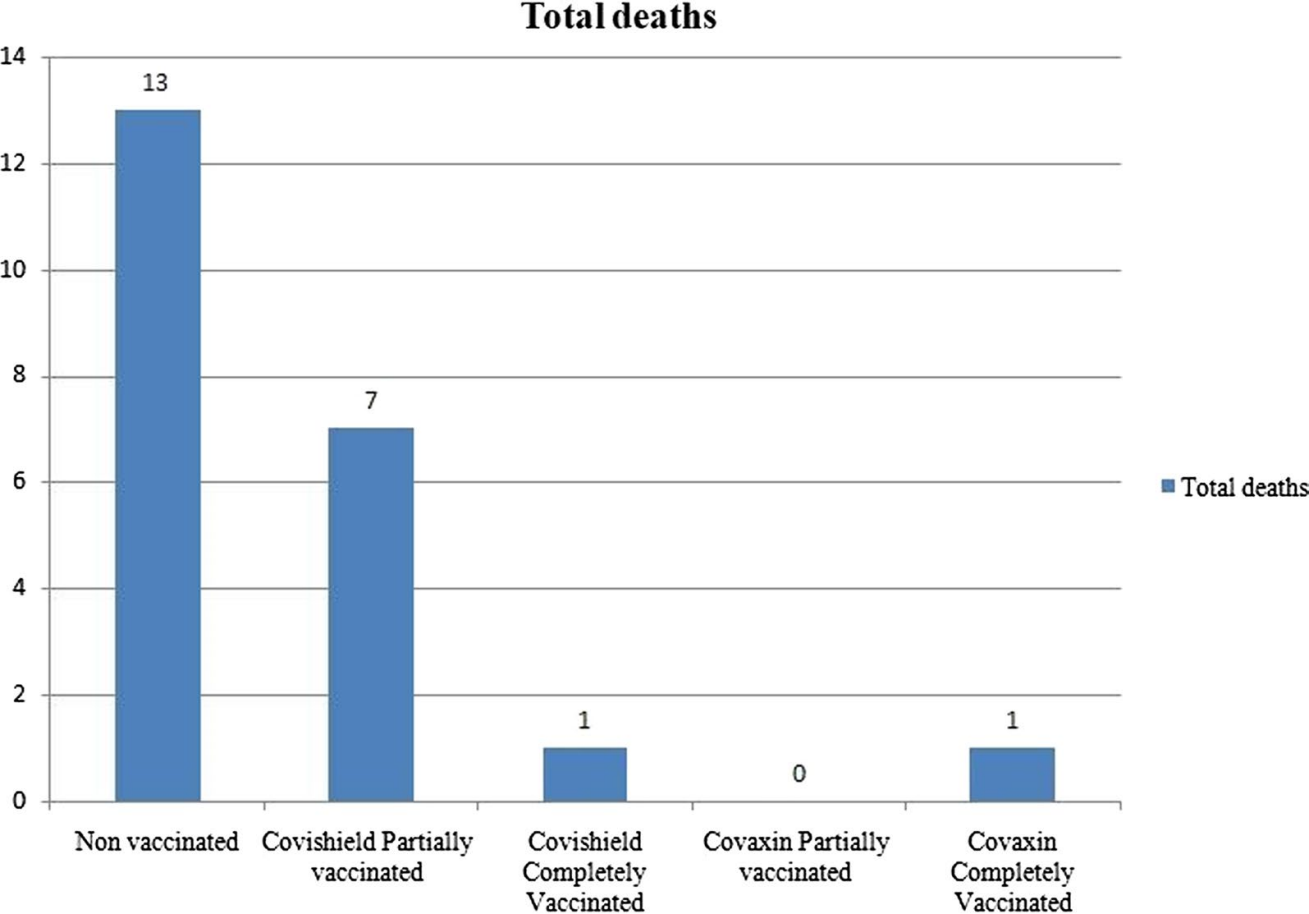
Mortality of the cohort

In terms of clinical outcome, 284 patients (92.8%) showed clinical improvement, and 22 patients (7.2%) died during the course of treatment in the hospital. Fourteen (66.6%) of the death were above the mean age of 62.56 ± 8.9 years, and males [*n*-17, (72.3%)] were more affected compared to females [*n*-5, (22.7%)]. The majority were associated with comorbidities [*n*-20, (90.9%)], and among them, 16 cases (72.7%) had multiple comorbidities. Out of 22 death, the CT severity was mild in three patients (13.7%), moderate in 13patients (59.0%), and severe in six patients (27.3%). The vaccination status among the dead is depicted in Fig. 6. The mortality was least among completely vaccinated group [*n*-2, (9.0%)] when compared to non-vaccinated group [*n*-13, (59.0%)] and partially vaccinated group [*n*-7, (31.0%)]. The least number of deaths was noted among the Covaxin recipients [*n*-1, (11.1%)] when compared to Covishield recipients [*n*-8, (88.9%)], irrespective of the number of doses. However, among the survivors, there was no statistically significant difference between the clinical outcome and vaccination status among the three groups [*χ*^2^ (2) = 3.228, *p* = 0.199].

## Discussion

Chest CT imaging has played a significant role in the ongoing COVID-19 pandemic. Initially, WHO had advised chest imaging as a part of the screening and diagnostic tool whenever RT-PCR testing was not available or wherever the test was delayed, or when there was a clinical suspicion of COVID-19 with initial negative RT-PCR testing [6]. Furthermore, CT chest imaging was used to assess the disease severity using a semi-quantitative CT severity scoring system and categorized into mild, moderate, and severe. The CT severity scoring system showed promising results as the CT scores correlated positively with the clinical stage and laboratory parameters of the disease [6, 9, 10]. As the CT imaging provides a direct objective assessment of the pulmonary parenchymal involvement compared with non-specific inflammatory biomarkers, it evaluates the disease burden and plays a vital role in the disease management, severity, and possible outcome [6, 10]. The study used the CT severity score as a potential imaging biomarker to evaluate the disease severity among vaccinated and non-vaccinated patients of COVID-19 pneumonia.

Covishield has shown the efficacy of 76.0% at preventing symptomatic COVID-19 following the first dose and 81.3% after the second dose. In contrast, Covaxin following two doses showed vaccine efficacy of 77.8% against symptomatic COVID-19 disease and the higher efficacy of 93.4% against severe COVID-19 disease. At the time of this writing, many studies have been done regarding the safety, immunogenicity, adverse effects, and protective effects of the vaccine concerning disease prevention, hospitalization, need for oxygen, and ICU admission [3, 4, 11, 12]. Victor et al. reported a protective effect of vaccination in preventing infection (65%), hospitalization (77%), need for oxygen (92%) and ICU admission (94%) [12]. Likewise, a study on 23,324 health care workers in the UK showed symptomatic and asymptomatic infections occurred in 80 participants (3.8%) among vaccinated and 977 (38%) among unvaccinated [13]. Our study revealed a positive protective effect of the vaccines using CT severity score as a biomarker of disease severity among symptomatic patients.

Our study also revealed a decrease in the LOS among vaccinated patients and further reduced LOS among Covishield recipients compared to Covaxin recipients. The potential predictors for prolonged LOS and increased risk of adverse complications include advanced age, higher levels of neutrophil counts and elevated inflammatory markers like D-dimer and CRP [14]. Apart from these, LOS also depends on other factors like admission and discharge criteria, bed demand and availability, and different timing within the pandemic [15].

The mortality rate of our study was 7.2%. Poor prognosis in our study was observed among men and patients with multiple comorbidities. These were consistent with previous reports [16–18]. The single-center study with a small sample size limited the interpretation of outcomes among the vaccinated population.

This is the first study to assess the protective effects of vaccination among symptomatic patients using CT severity score as an objective biomarker to the best of our knowledge. The study was done on the elderly population (above 50 years), and the majority had comorbidities (84.6%)—these high-risk groups concerning severe disease and mortality in the ongoing global pandemic.

Even though the study aims to objectively assess the efficacy of vaccines, a few limitations have been noted. Only a small cohort of patients, those above 50 years, was studied; the number was limited owing to a few factors like the age criteria for vaccine administration, demand–supply mismatch of the vaccines, resulting in reduced vaccination coverage. The timing of the first CT imaging varied among the cohort; as is known, the disease has a crescendo-decrescendo pattern, which could have contributed to variations in the estimation of moderate/severe disease.

To obtain a proper objective assessment, a multi-centric prospective case–control study with larger cohorts, including all age groups and imaging on a particular day of disease, will be necessary for more comprehensive results.

## Conclusions

The vaccine’s protective efficacy against severe SARS-COV2 infection could be validated through an objective assessment of pulmonary involvement using CT-SS. However, despite ramping up production of vaccines and ensuring an increase in accessibility across all age groups, the horizon appears to be grim as the pandemic of SARS-CoV-2 continues unabated, with newer variants posing a considerable threat.

Vaccines might be a game-changing tool and critical in ending the COVID-19 pandemic; however, studies evaluating vaccine efficacy in the occurrence of extra-pulmonary involvement secondary to COVID and long COVID are awaited.

## Data Availability

The datasets generated and/or analyzed during the current study are not publicly available due to privacy of the study participants.

## Abbreviations

CT: Computed tomography
COVID-19: Coronavirus disease 2019
HRCT: High resolution computed tomography
CT-SS: CT severity score
SARS-CoV-2: Severe acute respiratory syndrome coronavirus 2
MoH: Ministry of Health
TLR: Toll-like receptor
ICU: Intensive care unit
WHO: World Health Organization
PACS: Picture Archiving and Communication Systems
MDCT: Multi-detector computed tomography
LOS: Length of hospital stay
RT-PCR: Reverse-transcriptase polymerase chain reaction
